# The importance of using public data to validate reported associations

**DOI:** 10.1371/journal.pgen.1007416

**Published:** 2018-06-29

**Authors:** Georgia Chenevix-Trench, Jonathan Beesley, Paul D. P. Pharoah, Andrew Berchuck

**Affiliations:** 1 Queensland Institute of Medical Research Berghofer, Herston, Queensland, Australia; 2 Centre for Cancer Genetic Epidemiology, Department of Public Health and Primary Care, University of Cambridge, Strangeways Research Laboratory, Cambridge, United Kingdom; 3 Department of Obstetrics and Gynecology, Duke University Medical Center, Durham, North Carolina, United States of America; Cleveland Clinic Genomic Medicine Institute, UNITED STATES

We were interested to read the paper by Eng and colleagues on paternal lineage early onset hereditary ovarian cancers [1]. They found by exome sequencing of 159 non-*BRCA1/2* ovarian cancer cases that one missense variant (rs176026 in *MAGEC3*) was associated with risk at “chromosome-wide significance” (Hazard ratio [HR] = 2.85, 95% CI 1.75–4.65) and with a 6.7-year-earlier age of onset. They also report some in silico analyses of rs176026 and *MAGEC3*. However, other publicly available data on ovarian cancer risk for this SNP do not support these findings. In an analysis by the Ovarian Cancer Association Consortium (OCAC) [2], in over 25,000 ovarian cancer cases and 40,000 controls, no significant association was found for overall ovarian cancer risk (*P* = 0.24) or for any histological subtypes examined (*P* = 0.20 for high-grade serous ovarian cancer). In this data set, rs176026 was imputed with an imputation r-squared of 0.79.

We believe that it ought to be standard practice in an era of widespread data sharing for authors to identify relevant data that are publicly available and report and discuss their own findings in the context of the findings from such data. Relevant data would include data on the same or closely related phenotypes and on the same or highly correlated genetic variants.

In this instance, such data are publically available at http://ocac.ccge.medschl.cam.ac.uk/data-projects/results-lookup-by-region/, and the authors should have checked the findings in the OCAC data (to which they are contributors) and discussed its relevance to their own findings.
